# Total Phenolic, Total Flavonoids, Antioxidant and Antimicrobial Activities of *Scrophularia Striata* Boiss Extracts

**Published:** 2013-02-13

**Authors:** Mohaddese Mahboubi, Nastaran Kazempour, Ali Reza Boland Nazar

**Affiliations:** 1 Microbiology Group, Medicinal Plants Research Center of Barij Essence, Kashan, IR Iran; 2 Agricultural Group, Medicinal Plants Research Center of Barij Essence, Kashan, IR Iran

**Keywords:** Antimicrobial, Antioxidant, Phenolic Compounds

## Abstract

**Background:**

*Scrophularia striata* (Scrophulariaceae family) is an herbaceous plant that is traditionally used for treatment of microbial infections.

**Objectives:**

Antimicrobial and antioxidant activity of different extracts (methanolic, ethanolic, aqueous and ethyl acetate) from *S. striata* aerial parts was evaluated.

**Materials and Methods:**

The antimicrobial activity of different extracts from *S. striata* was evaluated against a large number of bacteria and fungi by micro broth dilution. Total phenolic and flavonoid contents were measured and their antioxidant activities evaluated by DPPH assay and beta carotene linoleic acid test.

**Results:**

Antimicrobial screening exhibited the positive relation between the total phenolic content and its antimicrobial activity but their antioxidant activity had a negative relation.

**Conclusions:**

Further studies are recommended against clinical isolate of sensitive bacteria and deep investigation on flavonoid and phenolic compounds of *S. striata* and detecting the antioxidant portion in aqueous extract.

## 1. Background

*Scrophularia striata*, an herbaceous flowering plant from Scrophulariaceae family, is commonly known as figwort. It has square stems, opposite leaves and open two-lipped flowers forming clusters at the end of their stems. The name *Scrophularia* comes from scrofula, a form of tuberculosis, since several species have been used to treat tuberculosis. It has been traditionally used for treatment of allergies, rheumatics and chronic inflammatory diseases. Some pharmacological activities of *S. striata* have been confirmed. *Scrophularia* extracts decrease edema, cell infiltration and proliferation of activated T-lymphocytes in joint tissues (1). Additionally, it inhibits a number of inflammatory factors such as PGE-2, leukotriene B4, NO, IL-1β, IL-4, INF-γ, but did not have any effect on the production of IL-10 (2).

Ethanolic extract of *S. striata* aerial parts acts as a nitric oxide inhibitor in a dose dependent manner in peritoneal macrophages and exhibited an anti-inflammatory effect (3). The antimicrobial activity of *S. striata* ethanolic extract leaves against *Staphylococcus aureus* and *Escherichia coli* (4), *Enterococcus faecalis* and Newcastle diseases (5) were confirmed. Additionally, this extract exhibited synergism activity with deoxycycline and of loxacin with FIC index 0.43 and 0.314, respectively (4). Ethanolic extract of *S. striata* had inhibitory effect against matrix metalloproteinase (MMP) (6). The leaves and seeds of *S. striata* contain both anticancer and cell growth enhancing activities (7).

## 2. Objectives

The purpose of this research is to evaluate the antimicrobial activity of different extracts from *S. striata* aerial parts against Gram positive, Gram negative bacteria and fungi. We also evaluated the total phenolic compounds in each extract and their antioxidant activity.

## 3. Materials and Methods

### 3.1. Plant Material and Extraction

The dried aerial parts of *Scrophularia striata* was collected from Kolm Valley (Ilam Province, Iran) in April 2010 and identified by Agriculture Department of Barij Essence Center, Kashan, Iran and authenticated under number 193-1. The aerial sample was ground and subjected to extraction by percolation method with water, methanol, ethyl acetate and ethanol-water (70:30, volume/volume). The powdered *S. striata* aerial parts were mixed with solvent at the ratio of 1:10 (w/v) in a percolator for 24 h at ambient temperature. Then, the mixture was filtered through Whatman filter paper No. 2, the residue rinsed with the same solvent and the extract dried under vacuum.

### 3.2. Total Phenolic Content (TPC)

Total phenolic contents of crude extracts were determined by a spectrophotometer using the Folin-Ciocalteu’s reagent (Merck) (8). A concentration 1 mg/mL of each extract was prepared. 0.1 mL of extract and 0.5 mL of Folin-Ciocalteu’s reagent (10%) was added. After 3-8 minute, 0.4 mL of 7.5% (w/v) sodium carbonate solution was added and mixed. Following 1 hour at ambient temperature, the absorbance of reaction mixture was measured at 765 nm. TPC was calculated using the following equation: W = ((Abs-0.0089)/0.0647) × 100, where Abs is absorbance and w is the weight (µg). All tests were conducted in triplicate and averaged. The results were expressed as mg of TPC per gram of dry extract as Gallic acid.

### 3.3. Total Flavonoid Content (TFC)

The modified aluminum chloride colorimetric method was used (9). 0.5 mL of diluted standard solution and each extract were separately mixed with 1.5 mL of ethanol (95%), 0.1 mL of aluminum chloride (10%), 0.1 mL potassium acetate (1 M) and 2.8 mL distilled water. Following incubation at room temperature for 30 minutes, the absorbance of the reaction mixture was measured at 415 nm with a spectrophotometer. The results were expressed as mg of TFC per gram of dry extract as Quercetin (QE).

### 3.4. Antioxidant Activity

#### 3.4.1. Free Radical Scavenging Activity by DPPH Method

Radical scavenging activity of the extracts was determined using DPPH radical scavenging activity assay (10) with a slight modification. A stock solution of each extract and BHT as the reference antioxidant (63 mg/1.6 mL) was prepared in its own solvent and diluted with methanol in the range of concentrations. 0.1 mL of diluted extract (7.8 to 2000 μg/mL) were added to 2 mL of freshly prepared DPPH methanol solution and mixed. After 30 minutes, the absorbance of reaction mixture was measured at 490 nm by spectrophotometer. Inhibition of free radical DPPH in inhibition percent (I%) was calculated and a sample concentration providing 50% inhibition (IC_50_) was calculated by plotting inhibition percentages against concentrations of the sample. All tests were carried out in triplicate and IC_50_ values were reported as means. BHT was used as positive control.

#### 3.4.2. β-carotene/ Linoleic Acid Bleaching Test (BCBT)

A diluted β-carotene in chloroform was transferred from pipette into a flask. Chloroform was removed using a rotary evaporator under vacuum at 50 °C for 10 minutes, and then 25 μl of linoleic acid, 200 mg of Tween 80 and 100 mL aerated distilled water were added to the flask with vigorous shaking. The emulsion (2.5 mL) was added to a tube containing 0.35 mL of the extract (2 mg/mL) and the absorbance immediately measured at 490 nm against a blank as zero time. Blank sample, devoid of β-carotene, was prepared for background subtraction. The tubes were placed in a water bath at 50 °C and the oxidation of the emulsion was monitored by measuring absorbance at 490 nm over a 120 minutes period. The antioxidant property (inhibition percentage, I%) of the samples was determined. BHT was used as positive control (11).

### 3.5. Microbial Strains and Antimicrobial Evaluation by Micro Broth Dilution Assay

*Staphylococcus aureus* ATCC 25923, *Staphylococcus epidermidis* ATCC 14490, *Staphylococcus saprophyticus* ATCC 15305, *Enterococcus faecium* ATCC 25778, *Enterococcus faecalis* ATCC 29212, clinical isolate of *Streptococcus agalactiae*, *Streptococcus pneumoniae* ATCC 33400, *Streptococcus mutans* ATCC 35668, *Streptococcus sobrinus* ATCC 27607, *Streptococcus sanguis* ATCC 10556, *Streptococcus salivarius* ATCC 9222, *Bacillus cereus* ATCC 1247, *Bacillus subtilis* ATCC 6051, *Klebsiella pneumoniae* ATCC 10031, *Escherichia coli* ATCC 8739, *Salmonella typhimurium* ATCC 14028, *Shigella dysenteriae* PTCC 1188, *Shigella flexeneri* NCTC 8516, *Enterobacter aerogenes* NCTC 10009, *Pseudomonas aeruginosa* ATCC 9027, and fungi *Candida albicans* ATCC 10231, *Candida glabrata* ATCC 90030, *Aspergillus flavus*, *Aspergillus niger* ATCC 16404, *Aspergillus parasiticus* ATCC 15517, were used. Bacterial suspensions were prepared in Brain Heart Infusion (BHI) (Merck) to a concentration of approximately 10^8^ CFU/mL using standard routine spectrophometric methods. Suspensions of fungi were made in sabouraud dextrose broth (10^6^ CFU/mL). Subsequent dilutions were made from the above suspensions, which were then used in the tests.

The minimum inhibitory concentration (MIC) and minimum lethal concentration (MLC) values of extracts were determined by micro broth dilution assay. The extracts were twofold serially diluted with 10% DMSO which contains 25.6-0.2 mg/mL of each extract. Antibiotics were used as positive control. These dilutions were prepared in a 96-well microtitre plate. MOPS-buffered RPMI 1640 (fungi) (12), Cation adjusted Muller Hinton broth (non fastidious bacteria) (13) and Todd Hewitt broth (fastidious bacteria) (14) were used as broth media. After shaking, 100 µl of extract dilutions was added to each well. The above microbial suspensions was diluted (1 × 10^6^ CFU/mL for bacteria; 10^4^ CFU/mL for fungi) and then 100 µl was added to each well and incubated at 35 ºC. MICs were defined as the lowest concentration of extract dilutions that inhibits bacteria and fungi after 24, 48 hours, respectively. MLC values were the first well that showed no growth on solid media.

## 4. Results

The antimicrobial activity of *S. striata* extracts against different microorganisms was calculated by micro broth dilution assay (Table 1). The antimicrobial activity of *S. striata* ethanolic extract against *S. aureus, S. saprophyticus, S. epidermidis*, oral *Streptococcus sp* (*S. mutans, S. sobrinus, S. sanguis*), *Candida sp* (*C. albicans, C. glabrata*) and *A. parasiticus* exhibited higher activity than the other extracts. Among the Gram positive ones, *S. saprophyticus* had the low MIC and MLC values (1.6 and 3.2 mg/mL) to *S. striata ethanolic* extract. *S. sanguis* was the most sensitive *Streptococci* to ethanolic extract (MIC and MLC value 3.2 mg/mL). *S. striata* ethanolic extract had microbicidal activity against *S. pneumonia, S. sanguis, S. salivarius, B. subtilis, C. albicans* and *C. glabrata. S. striata* aqueous extract showed lower antimicrobial activity than that of other extracts. *S. epidermidis, S. sobrinus, K. pneumonia, B. subtilis* and *B. cereus* had more sensitivity to aqueous extract (MIC, MLC = 6.4 and 12.8 mg/mL). The activity of *S. striata* extracts on *S. pneumonia* was ethanolic extract > methanolic extract > ethyl acetate extract > aqueous extract.

**Table 1:** Antimicrobial Activity of *Scrophularia striata* Boiss Extracts by Microbroth Dilution Assay

	Water		Hydroalcoholic		Ethyl acetate		Methanolic		Antibiotic	
	MIC	MLC	MIC	MLC	MIC	MLC	MIC	MLC	MIC	MLC
***S. aureus***	12.8	12.8	3.2	6.4	6.4	12.8	6.4	12.8	0.25	0.5
***S. saprophyticus***	12.8	12.8	1.6	3.2	6.4	12.8	6.4	12.8	2	4
***S. epidermidis***	6.4	12.8	3.2	6.4	6.4	12.8	6.4	12.8	0.5	1
***S. pneumoniae***	25.6	25.6	0.4	0.4	6.4	6.4	0.4	0.8	0.25	0.5
***S. agalactiae***	25.6	25.6	25.6	25.6	12.8	12.8	12.8	25.6	0.5	0.5
***E. faecalis***	25.6	25.6	25.6	25.6	12.8	12.8	12.8	25.6	1	2
***E. faecium***	25.6	25.6	25.6	25.6	6.4	12.8	12.8	25.6	1	2
***S. mutans***	12.8	12.8	3.2	12.8	6.4	128	12.8	12.8	2	4
***S. sobrinus***	6.4	12.8	3.2	6.4	6.4	64	12.8	12.8	0.5	1
***S. sanguis***	12.8	12.8	3.2	3.2	6.4	6.4	12.8	12.8	0.25	0.5
***S. salivarius***	12.8	12.8	12.8	12.8	6.4	12.8	12.8	12.8	1	2
***Sh. flexeneri***	12.8	12.8	3.2	12.8	6.4	12.8	6.4	25.6	4	4
***E. coli***	12.8	25.6	6.4	6.4	25.6	25.6	6.4	25.6	0.5	1
***S. typhimurium***	25.6	25.6	6.4	25.6	25.6	25.6	6.4	25.6	1	1
***K. pneumoniae***	6.4	12.8	6.4	12.8	25.6	25.6	6.4	25.6	0.25	0.25
***Sh. dysenteriae***	25.6	25.6	3.2	12.8	25.6	25.6	3.2	25.6	0.125	0.125
***P. aeruginosa***	25.6	25.6	6.4	6.4	25.6	25.6	64	12.8	1	1
***E. aerugenes***	25.6	25.6	6.4	12.8	25.6	25.6	6.4	25.6	0.25	0.5
***B. subtilis***	6.4	128	3.2	3.2	0.8	1.6	0.8	6.4	0.125	0.125
***B. cereus***	6.4	128	6.4	12.8	6.4	12.8	6.4	6.4	0.25	0.5
***C. albicans***	25.6	25.6	6.4	6.4	12.8	25.6	3.2	25.6	0.125	0.125
***C.glabrata***	25.6	25.6	6.4	6.4	12.8	25.6	6.4	25.6	0.06	0.06
***A. flavus***	25.6	25.6	6.4	25.6	12.8	25.6	12.8	25.6	0.25	0.25
***A. parasiticus***	25.6	25.6	6.4	12.8	12.8	25.6	12.8	25.6	0.125	0.125
***A. niger***	25.6	25.6	6.4	25.6	12.8	25.6	12.8	25.6	0.125	0.25

*S. striata* methanolic extract on different gram negative ones excluding *P. aeruginosa*, filamentous fungi and yeast was inhibitory effect. Antimicrobial effect of *S. striata* extracts on filamentous fungi is weak and only its ethanolic extract was effective against *C. albicans, C. glabrata* and *A. parasiticus*. Among gram negative ones, *E. coli* and *P. aeruginosa* showed more sensitivity to ethanolic extract and *P. aeruginosa* to methanolic extract. Total phenolic and flavonoid content of *S. striata* extracts were evaluated. The results have been revealed in Table 2. Total phenolic content of ethanolic extract (79.7 mg GAC/g), ethyl acetate (65.5 mg GAC/g) was higher than that of methanolic extract (49.1 mg GAC/g) and aqueous extract (36.6 mg GAC/g). The total flavonoid content of ethyl acetate extract (27.5 mg QE/g) was higher than ethanolic extract (9.8 mg (QE/g), methanolic extract (8.2 mg QE/g) and aqueous extract (5.1 mg QE/g). Ethyl acetate extract had higher total flavonoid content and ethanolic extract had higher total phenolic content. *S. striata* water extract had the lower phenolic and flavonoid content than the other extracts. The amount of phenolic content in methanolic extract was lower than ethanolic or ethyl acetate extract however its flavonoid content was a small lower than ethanolic extract.

Antioxidant activity of *S. striata* extracts was evaluated by DPPH and BCBT assays (Table 2). In DPPH assay, IC_50_ of extracts revealed that *S. striata* aqueous extract with IC_50_ 195 µg/mL had higher antioxidant activity. The IC_50_ of methanolic and ethanolic extracts was the same. Ethyl acetate extract had higher IC_50_. The antioxidant activity of *S. striata* was weaker than BHT (Table 2). In BCBT assay, evaluation of inhibition percent (I%) showed that aqueous, methanolic, ethanolic and ethyl acetate extracts had I% 70.2%, 68%, 64.8% and 58.3%, respectively.

**Table 2:** Total Phenolic Content (TPC), Total Flavonoid Content (TFC) and Antioxidant Capacity of the Extracts Tested in this Study

Solvent	TPC, mg/g	TFC, mg/g	IC_50_, µg/mL	BCBT,%I
**Water**	34.6	5.1	195	70.2
**Methanol**	49.1	8.2	320	68
**Ethanol 70%**	79.7	9.8	240	64.8
**Ethyl acetate**	65.5	27.5	2000	58.3
**BHT**	-	-	20	72.6

## 5. Discussion

Oxygen and nitrogen reactive species are essential for energy storage, toxification, chemical signaling and immunological functions also they control endogenous enzymes like superoxide dismutase, glutathione peroxidase and catalase. Failure in defense mechanisms is the agent of producing oxygen's free radicals that can disturb essential molecules such as DNA, lipids and proteins. These injuries are associated with cardiovascular diseases, cancer and other chronic diseases.

Natural antioxidants can protect body against oxidative injuries and decrease the risk of different chronic diseases (15). This hypothesis has been reached by the researcher who evaluated the relation between natural antioxidant in medicinal plants and the decrease in degenerative diseases. Medicinal plant's antioxidants such as phenolic represent a large group of antioxidant compounds. Phenolic compounds are able to control enzymatic activity and different clusters of herbal phenolic such as polyphenols are able to attach to proteins and this attachment causes the further attachment to cellular transporters and receptors. Flavonoids are a common cluster of phenolic compounds that can synthesize from phenyl alanine amino acid.

From the above explanation, we expected that ethanolic or ethyl acetate extracts with higher level of total phenolic content would exhibit higher antioxidant activities. Although many studies exhibit that there are a positive relation between total phenolic content and their antioxidant activities (16), our research demonstrates that there is no positive relation between total phenolic content of *S. striata* extracts and its antioxidant activity. The *S. striata* aqueous, methanolic and ethanolic extracts had higher antioxidant activity than ethyl acetate extract. The result showed a higher flavonoid content of ethyl acetate extract than of others, and that this amount is the same in ethanolic and methanolic extracts. It was expected that ethyl acetate extract would have the higher antioxidant activity than that of other extracts but surprisingly the aqueous extract has the higher antioxidant activity. Since the natural antioxidant include many different compounds such as phenol, nitrous compounds, caroteonids and many unidentified compounds (17), compounds other than the phenolic compounds may be responsible for antioxidant activity in water, methanolic or ethanolic extract or sometimes the structure of flavonoids in ethyl acetate extract may decrease its antioxidant activity. Rice-Evans et al (18) showed that hydroxyl groups, the amount of conjugation and its kind, are two important factors in antioxidant potential of phenolic compounds. Stronger antioxidants usually are more conjugated and have more hydroxyl groups that make the antioxidant strong enough to scavenge the free radicals. Further study is needed for demonstration of this issue.

*S. striata* is traditionally used for treatment of infectious diseases. The use of *S. striata* in treatment of infectious diseases has important role in reducing of chemical's side effect and breaking the antimicrobial resistance. The result of this study has been shown that the antimicrobial activity of *S. striata* is depended on the type of microorganism. The result of antimicrobial activity of *S. striata* ethanolic extract against *S. aureus* is coincidental to other studies (4). *P. aeruginosa* is resistant gram negative bacteria to many antibiotics, so its sensitivity to methanolic and ethanolic extract of *S. striata* is an important issue for finding the effective fraction against this resistant bacterium. Ethanolic extract from aerial parts of *S. striata* have higher antimicrobial activity than that of aqueous extract, although its flavonoid content is lower than methanolic or ethyl acetate extract. However, its phenolic content is higher than that of others in that it exhibits the positive relation between the total phenolic content and its antimicrobial activity. Further study is recommended against clinical isolate of sensitive bacteria and deep investigation on flavonoid and phenolic compounds of *S. striata* and identifying the antioxidant portion in aqueous extract.
